# A decrease in NR2B expression mediated by DNA hypermethylation induces perioperative neurocognitive disorder in aged mice

**DOI:** 10.1111/cns.14097

**Published:** 2023-01-24

**Authors:** Feifei Xu, Peilin Cong, Bingqian Zhang, Hailong Dong, Wenqiang Zuo, Tingmei Wu, Li Tian, Lize Xiong

**Affiliations:** ^1^ Department of Anesthesiology and Perioperative Medicine Xijing Hospital, Fourth Military Medical University Xi'an China; ^2^ Department of Anesthesiology and Perioperative Medicine Shanghai Fourth People's Hospital Affiliated to Tongji University School of Medicine, Translational Research Institute of Brain and Brain‐Like Intelligence Affiliated to Tongji University School of Medicine, Shanghai Key Laboratory of Anesthesiology and Brain Functional Modulation Shanghai China; ^3^ Shanghai Key Laboratory of Psychotic Disorders, Shanghai Mental Health Center Shanghai Jiao Tong University School of Medicine Shanghai China

**Keywords:** DNA methylation, NR2B, perioperative neurocognitive disorder, SAM

## Abstract

**Aims:**

This study was designed to investigate the role of NR2B and the contribution of DNA methylation to NR2B expression in the pathogenesis of PND.

**Methods:**

Eighteen‐month‐old C57BL/6J mice were subjected to experimental laparotomy under 1.4% isoflurane anesthesia. Hippocampus‐dependent learning and memory were evaluated by using the Barnes maze and contextual fear conditioning tests. The protein and mRNA expression levels of NR2B were evaluated by western blotting and qRT–PCR respectively, and the methylation of the *NR2B* gene was examined by using targeted bisulfite sequencing. Long‐term synaptic plasticity (LTP) was measured by electrophysiology.

**Results:**

Mice that underwent laparotomy exhibited hippocampus‐dependent cognitive deficits accompanied by decreased NR2B expressions and LTP deficiency. The overexpression of NR2B in the dorsal hippocampus could improve learning and memory in mice subjected to laparotomy. In particular, the decreased NR2B expressions induced by laparotomy was attributed to the *NR2B* gene hypermethylation. Preoperative administration of S‐adenosylmethionine (SAM) could hypomethylate the *NR2B* gene, upregulate NR2B expression and improve LTP, exerting a dose‐dependent therapeutic effect against PND. Moreover, inhibiting NR2B abrogated the benefits of SAM pretreatment.

**Conclusions:**

Laparotomy cause hippocampus‐dependent cognitive decline by hypermethylating the *NR2B* gene, allowing us to understand the pathogenesis of PND in an epigenetic landscape.

## Graphical abstract

A decrease in NR2B expression mediated by DNA hypermethylation contributes to the pathogenesis of PND in aged mice, and SAM can function as a “hypomethylating” agent to hypomethylate the *NR2B* gene, rather than a pure “hypermethylating” agent as it used to be predicted, exerting a dose‐dependent therapeutic effect against PND.

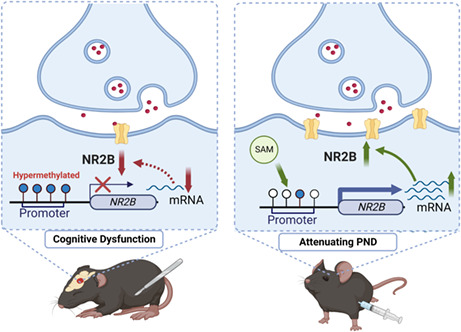

## 1 INTRODUCTION

Perioperative neurocognitive disorder (PND) is a syndrome characterized by impaired cognitive performance on a battery of neuropsychological tests during the perioperative period, including transient postoperative delirium and longer‐lasting deficits in concentration, memory and learning especially in aged patients.^1^ The incidence of PND depends on perioperative and intraoperative patient‐related risk factors and may even reach 53.3% in hip fracture patients. PND not only diminishes the quality of life but also threatens the lives of patients, imposing a significant social and economic burden.^2^ Unfortunately, there is still a lack of effective therapeutic approaches.

*N*‐methyl‐D‐aspartate (NMDA) receptors are major excitatory glutaminergic receptors expressed in the nervous system. Functional NMDARs are heterotetrameric complexes composed of NR1 and NR2 (NR2A‐D) or NR3 (NR3A‐B) subunits.^3^ Among these subunits, the NR2B subunit, which is encoded by the gene *NR2B* or *Grin2b*, is crucial in determining the physiological and molecular properties of NMDA receptors, particularly in relation to synaptic plasticity and cognitive ability.^4^, ^5^ It has long been known that the overexpression of NR2B enhances LTP and improves learning and memory in both adult and aged animals, whereas NR2B knockout mice display LTP and learning impairment in the Morris water task and contextual fear conditioning test.^4^, ^6^, ^7^ However, the role of NR2B in PND is not fully understood, and how NR2B expression is regulated by laparotomy is largely unknown.

Over the past few decades, epigenetic programming has emerged as an important mechanism underlying alterations in gene function upon exposure to environmental stimuli without a germline alteration in DNA sequence. DNA methylation is a well‐known epigenetic mechanism that is preferentially distributed over nucleosome regions in chromatin and is typically associated with gene silencing by interfering with chromatin accessibility for *cis* regulatory elements (e.g. transcription factors).^8^ An imbalance of DNA methylation has been regarded as a valuable biomarker for aging and aging‐related pathologies, including PND.^9^, ^10^, ^11^ Importantly, previous studies have reported that methylation at specific CpG sites around the promoter of *NR2B* gene will downregulate *NR2B* transcription.^12^, ^13^ However, whether DNA methylation is involved in the regulation of NR2B by laparotomy and the associated cellular mechanism is still unknown.

Therefore, the present study first examined the role of NR2B in hippocampal‐dependent learning and memory deficits induced by laparotomy, and then investigated the effects of laparotomy on *NR2B* methylation. Furthermore, we explored the contribution of *NR2B* methylation to NR2B expression during the occurrence of PND by modulating SAM, a methylome modulator, and evaluated the therapeutic effects of SAM against PND. This would provide a better understanding of the pathogenesis of PND and novel therapeutic targets for the prevention and treatment of PND.

## 2 MATERIALS AND METHODS

### 2.1 Animals and experimental design

To avoid the variability thought to be caused by estrous cycle,^14^ only male mice were used in this study. Eighteen‐month‐old mice are used because we previously found that using 18 months old mice that correspond to ~60 year old humans to establish PND model is more stable.^15^, ^16^, ^17^, ^18^ C57BL/6J mice were housed in standard cages under controlled laboratory conditions (23 ± 1°C, 50% humidity, 12‐h light–dark cycle [lights on from 7 a.m. to 7 p.m.]) with ad libitum access to food and water. All behavioral tests were performed during the light phase.

In the first experiment, mice were randomly assigned to control group (not being exposed to laparotomy) and laparotomy group (being subjected to laparotomy). The animals were used for neurocognitive function assessment starting 3 days after the laparotomy (*n* = 8). Hippocampus were harvested for RNA sequencing (*n* = 3) 1 day after laparotomy, and for western blotting (*n* = 3) or electrophysiological recording (*n* = 4) 3 days after laparotomy.

In the second experiment, mice were randomly assigned to lentivirus control group (*rLV‐Con* group) and lentivirus *NR2B* overexpression group (*rLV‐NR2B* group). Mice received a bilateral dorsal hippocampal injection with *rLV‐hSyn‐EGFP‐WPRE* (7 × 10^8^ TU/mL) or *rLV‐hSyn‐NR2B‐EGFP‐WPRE* (6 × 10^8^ TU/mL). Three weeks after the lentivirus injection, mice were subjected to laparotomy. Hippocampi were harvested for western blotting (*n* = 3) 3 days after laparotomy (*n* = 3) and the animals were used for neurocognitive function assessment starting 3 days after laparotomy (*n* = 8). One animal of the *rLV‐NR2B* group died 5 days after laparotomy and was not considered.

In the third experiment, mice were randomly assigned to the control group and the laparotomy group. Hippocampi were harvested for qRT**–**PCR (*n* = 6) and targeted bisulfite sequencing (*n* = 3) 3 days after laparotomy.

In the fourth experiment, mice were randomly assigned to the control group and the laparotomy group. The concentrations of homocysteine (Hcy), SAM, and s‐adenosylhomocysteine (SAH) in hippocampus were measured 1, 3, and 7 days after laparotomy (*n* = 5).

In the fifth experiment, mice were randomly assigned to the saline group (being subjected to laparotomy 20 min after saline administration) and the SAM group (being subjected to laparotomy 20 min after 100 mg/kg SAM administration). Hippocampi were harvested for targeted bisulfite sequencing (*n* = 3), qRT**–**PCR (*n* = 5), western blotting (*n* = 3) and electrophysiological recording (*n* = 4) 3 days after laparotomy.

In the sixth experiment, mice were randomly assigned to (1) the saline group (mentioned above) and (2) to (4) groups (being subjected to laparotomy 20 min after SAM (25, 50, 100 mg/kg) administration). The animals were used for neurocognitive function assessment starting 3 days after laparotomy (*n* = 8).

In the seventh experiment, mice were randomly assigned to (1): the saline group (mentioned above), (2) the SAM group (being subjected to laparotomy 20 min after 100 mg/kg SAM administration), (3) the SAM + ifenprodil group (being subjected to laparotomy 20 min after 100 mg/kg SAM administration, followed by 10 mg/kg ifenprodil administration three times), and (4) the SAM++RO25‐6981 group (being subjected to laparotomy 20 min after 100 mg/kg SAM administration, followed by 10 mg/kg RO25‐6981 administration three times). The animals were used for neurocognitive function assessment starting 3 days after laparotomy (*n* = 8).

### 2.2 Experimental laparotomy

A PND model was constructed by experimental laparotomy as reported previously.^15^ Briefly, mice were anesthetized by the inhalation of 2.0% isoflurane (Baxter Healthcare) for 2 min, and anesthesia was maintained with 1.4% isoflurane in 100% O_2_ at a rate of 1 L/min.^19^, ^20^, ^21^ Mice were placed on a heated surgical table to maintain the core body temperature at 37.5°C. After the abdominal hair was shaved and the surgical area was thoroughly disinfected with iodine, an approximately 1.5‐cm midline abdominal incision was made, and the muscle layers were separated along the ventral white line. A 5‐cm segment of the small intestine was gently pulled out from the surgical cavity and wrapped in gauze moistened with sterile saline. Subsequently, the small intestine was gently kneaded for 10 min to mimic clinical exploratory laparotomy. After the intestine was returned to the abdomen, the muscle and skin were closed layer by layer using 4–0 absorbable sutures (VICRYL; Ethicon, USA). Finally, topical EMLA cream (2.5% lidocaine and 2.5% prilocaine) was used for postoperative analgesia. The surgical procedure was performed under sterile conditions and lasted around 30 min. Finally, mice were allowed to recover in an incubator at 37°C for 30 min and then returned to their home cages.

### 2.3 Neurocognitive function assessment

#### 2.3.1 Open‐field test

Each mouse was gently placed in an open field arena composed of a 40 × 40 × 40 cm plastic box and allowed to explore freely for 5 min. All traces were recorded with a camera. The average speed (m s^−1^) and the time spent in the central area were quantified blindly using Smart Video Tracking Software (Panlab; Harvard Apparatus) to assess motor ability and anxiety, respectively.

#### 2.3.2 Barnes maze test

Hippocampus‐dependent spatial learning and memory were assessed through the Barnes maze paradigm performed as described previously.^15^ Briefly, the mice were allowed to explore an elevated circular platform (90 cm × 90 cm) with 20 equidistant holes around the periphery. On Day 3 after laparotomy, the mice were allowed to habituate to the maze for 3 min after white light (200 W) and white noise (85 dB) were applied, and they were then guided slowly toward the escape compartment. On the training days, the aversive stimuli were applied, and the mice were allowed to explore the maze for 3 min until they entered the escape compartment. Training trials were performed repeatedly for 4 consecutive days (3 trials per mouse on day 4 and 4 trials per mouse on days 5–7 after laparotomy). After training, the escape compartment was removed, and the mice were allowed to explore the maze for 2 min. To eliminate odor cues, the apparatus was carefully cleaned with 75% ethanol between each test. The time required to enter the escape compartment and the percentage of time spent in the target quadrant in a 2‐min period were calculated to assess spatial reference learning and memory. All data were recorded with a camera and analyzed blindly with Smart Video Tracking Software (Panlab; Harvard Apparatus).

#### 2.3.3 Contextual fear conditioning

Mice were subjected to the hippocampus‐dependent contextual fear conditioning test using the Ugo Basile Fear Conditioning System (Ugo Basile Srl) as described previously.^22^ On the day of habituation (day 8), the mice were allowed to move freely in the conditioning chamber (17 cm × 17 cm × 25 cm) for 10 min. On the day of training (day 9), the mice were placed in the chamber, and five foot shocks were delivered (current: 0.7 mA, 2 s; interval: 35–60 s). Then, the mice were returned to their home cages. Twenty‐four hours later, the mice were put into the same chamber for 5 min to assess contextual memory retrieval. Freezing was defined as no movement for 2 s. To eliminate odor cues, the apparatus was carefully cleaned with 75% ethanol between each test. The freezing time of each animal was analyzed blindly with ANY‐maze Software (Stoelting Co.).

### 2.4 RNA sequencing

Total RNA was extracted using an RNAeasy Mini kit (Qiagen). After library construction, sequencing was carried out with the Illumina NovaSeq 6000. CASAVA was used to assess the quality of the sequencing data; then, clean reads were aligned to the mouse genome (GRCm38/mm10) using Hisat2 (v2.0.5). FeatureCounts (v1.5.0‐p3) was used to count the reads numbers mapped to each gene, and FPKM of each gene was calculated. DEGs were determined using DEGseq (v1.34.0) and were defined as fold change (FC)>1.5 and FDR < 0.05.

### 2.5 SDS–PAGE and immunoblotting

Mice were anesthetized and transcardially perfused with 0.9% saline. Hippocampal tissues were carefully dissected and then lysed in RIPA buffer (Thermo Fisher) containing 1 mM phosphatase inhibitor, 1 mM protease inhibitor, and 1 mM PMSF (Beyotime Biotechnology) with an ultrasonicator. The proteins were separated on 7.5% SDS–PAGE gels and then transferred onto a polyvinyl difluoride (PVDF) membrane (Millipore). The membrane was blocked with 5% nonfat milk for 2 h at room temperature and then incubated with primary antibodies overnight at 4°C followed by an HRP‐linked anti‐rabbit IgG antibody (31466, 1:3000, Thermo Fisher) for 1 h at room temperature. The primary antibodies used were anti‐β‐actin (AF7018, 1:3000, Affinity) and anti‐NR2B (AF6426, 1:1000, Affinity). The target protein was detected by a ChemiDoc XRS+ system (Bio‐Rad) and then analyzed with ImageJ software (NIH).

### 2.6 Electrophysiological recording

Hippocampal slices were prepared from 18‐month‐old mice. Briefly, coronal slices containing the hippocampus (400 μm) were cut with a vibratome slicer (VT1200S, Leica) and incubated in artificial cerebrospinal fluid (ACSF) for at least 1 h before use. A single slice was transferred to the perfusion‐type recording chamber and visualized using infrared‐differential interference contrast microscopy. A concentric bipolar electrode (FHC, Lot#300125) was positioned in the stratum radiatum of CA1 to stimulate the afferent Schaffer collateral‐commissural pathway from the CA3‐CA1 region. Then, fEPSPs were recorded in the CA1 region using micropipettes and LTP was induced by delivery of HFS (100 Hz, 20‐s interval, four trains), the amplitude of the fEPSPs was usually set at 40–50% of the maximal responses. Data were recorded with a MultiClamp 700B (Axon instruments) and acquired with Clampex 10.7.

### 2.7 Stereotaxic injection

Recombined *rLV‐hsyn‐EGFP‐WPRE* vector with the *NR2B* (NM_008171.4) gene (*rLV‐NR2B*) or *rLV‐hsyn‐EGFP‐WPRE* with a scrambled control sequence (*rLV‐Con*) was constructed by GeneChem Company and bilaterally injected into the dorsal hippocampus (AP: −1.50 mm; ML: ±1.70 mm; DV: −1.75 mm) at a speed of 200 nL/min. The syringe was left for an additional 10 min in place before the incision was sutured. Laparotomy was performed 3 weeks later.

### 2.8 Quantitative reverse‐transcription PCR (qRT–PCR)

Total RNA was extracted using TRIzol reagent (Thermo Fisher) and reverse transcribed into cDNA using the PrimeScript RT reagent kit (Vazyme, China). Then, qRT–PCR was performed using SYBR Green Master Mix (Vazyme, China) on a Quant Studio 1 Real Time PCR system (Thermo Fisher, USA). *GAPDH* was used as a control. The following primers were used: *NR2B*, 5′‐ GCCATGAACGAGACTGACCC‐3′ and 5′‐ GCTTCCTGGTCCGTGTCATC‐3′; *GAPDH*: 5′‐AGGTCGGTGTGAACGGATTTG‐3′ and 5′‐GGGGTCGTTGATGGCAACA‐3′. The 2^−ΔΔ^Ct method was employed to assess the relative expression levels of genes of interest.

### 2.9 Targeted bisulfite sequencing

The BisPCR^2^ method, including bisulfite conversion of genomic DNA and two rounds of PCR for target enrichment and sample barcoding, was performed as described in a previous study with minor modifications.^23^ Specifically, hippocampal tissues were incubated with DNA lysis buffer (10 mM Tris (pH 8.0), 100 mM NaCl, 10 mM EDTA pH (8.0), 10% SDS) containing proteinase K at 37°C overnight, and DNA was extracted with the phenol–chloroform method. Then, the DNA concentration was quantified with a NanoDrop2000 spectrophotometer. Genomic DNA was bisulfite converted using an EZ DNA Methylation‐Gold™ Kit (Zymo) according to the manufacturer's instructions, and 100 ng bisulfite‐converted DNA was amplified using EpiTaq HS DNA polymerase (Takara) with primers targeting the *NR2B* gene (PCR #1), followed by a subsequent round of PCR for the amplification of pooled PCR #1 template with Phusion DNA polymerase (NEB) (PCR #2). The primers used for PCR #1 and PCR #2 are listed in Table S1. Barcoded libraries were pooled together and purified with 1.5× AMPure XP beads (Beckman Coulter). Finally, sequencing of 2× 150‐bp paired‐end reads was carried out on an Illumina Nova‐seq 6000 instrument.

### 2.10 Determination of Hcy, SAM, and SAH

Mice were anesthetized and perfused with 0.9% saline before hippocampal tissues were dissected. The concentrations of Hcy, SAM, and SAH in the hippocampus of mice were measured by using commercially available ELISA kits (Jianglai Biological Company) according to the manufacturer's instructions. Each sample was analyzed in triplicate.

### 2.11 Pharmacological treatment

Mice were randomly grouped and administered different doses (0, 25, 50, 100 mg/kg) of SAM (Macklin Biochemical Co., Ltd, China) by intraperitoneal injection 20 min before the anesthesia.^24^ An equal volume of saline was used as a control. For the NR2B intervention experiment, ifenprodil (Sigma–Aldrich) or RO25‐6981 (Med Chem Express) was dissolved in saline and intraperitoneally administered at a dose of 10 mg/kg three times at 48‐hour intervals starting immediately after the laparotomy.^25^, ^26^

### 2.12 Statistical analysis

The data are shown as the mean ± S.D. with the presentation of data of each individual animal. The sample size for each experiment was described above. We did not exclude any data. All the assays were repeated at least 3 times. Shapiro–Wilk test was used to check whether the data were normally distributed. Student's *t* test (two groups), one‐way ANOVA followed by Dunnett's post‐hoc test (multiple groups) and repeated measures two‐way ANOVA followed by Sidak's post‐hoc test (multiple groups at different time point) were used to analyze the data using GraphPad Prism 8.0 software (GraphPad Prism Co., USA). Data that did not exhibit a normal distribution were analyzed via Kruskal–Wallis test (multiple groups). *p* < 0.05 was considered significant.

## 3 RESULTS

### 3.1 Association of NR2B expression with laparotomy‐induced cognitive decline

**FIGURE 1:**
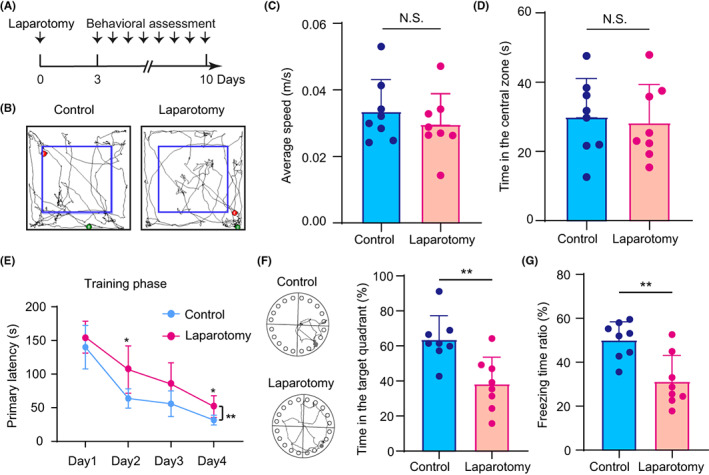
Laparotomy induced cognitive decline in 18‐month‐old mice. (A) Schematic timeline. (B‐D) Representative tracks (B), average speed (C), and time spent in the central zone (D) of animals in the open field tests. (E) Mean latency of animals in the training phase of the Barnes maze tests (4 consecutive days). (F) Representative tracks (left) and time spent in the target quadrant (right) of animals in the probe phase of the Barnes maze tests. (G) Ratio of freezing time of animals in the contextual fear conditioning tests. The data are presented as the mean ± SD and analyzed by two‐way ANOVA or Student's *t* test. N.S.: no significant difference, *p < 0.05, **p < 0.01.

As shown in Figure 1A–D, there were no significant differences in average speed or time spent in the central zone in the open field test between mice that underwent laparotomy and the control mice. However, in the Barnes maze test, the primary latency in the training phase was prolonged, and the time spent in the target quadrant in the probe phase was shortened (Figure 1E; 38.33% [15.25%] vs. 63.69% [13.60%], *p* = 0.0035, Figure 1F). In addition, mice that underwent laparotomy exhibited a decreased ratio of freezing time in the contextual fear conditioning test (31.20 [11.87] vs. 50.07 [8.338], *p* = 0.0025, Figure 1G). These results suggest that laparotomy induces severe cognitive impairment in aged mice.

**FIGURE 2:**
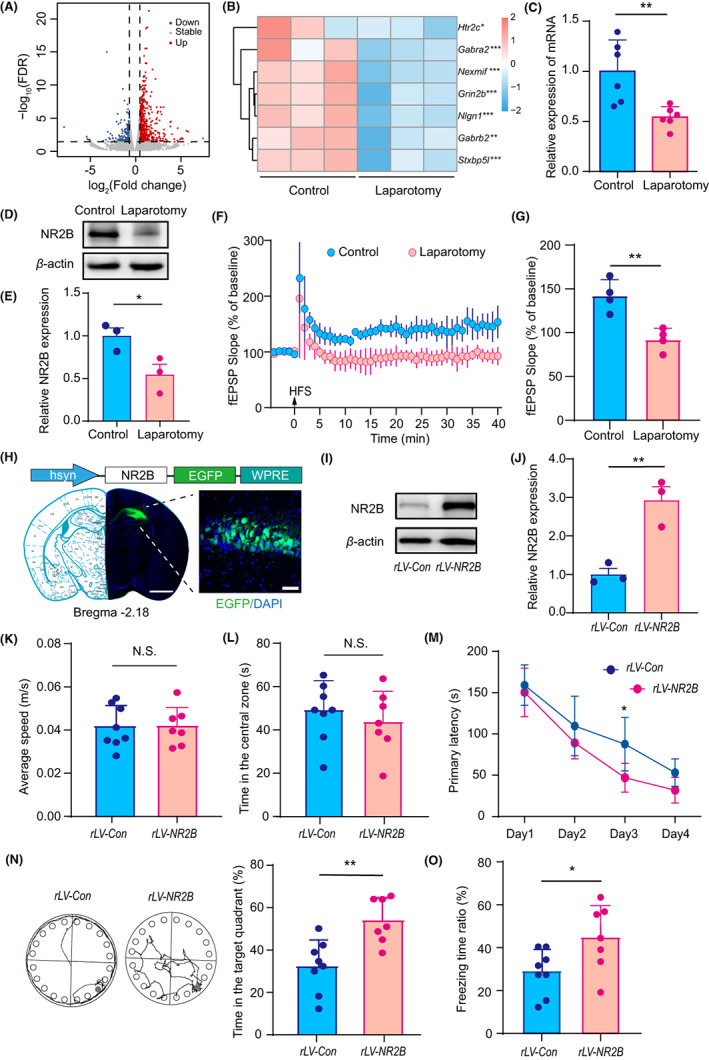
Association of NR2B expression with laparotomy‐induced cognitive decline in 18‐month‐old mice. (A) Volcano plot of DEGs. Genes with differential expression were defined by FDR <0.05 and a fold change >1.5. (B) Heatmap indicating seven genes that were significantly downregulated by laparotomy. (C) Levels of NR2B mRNA in the hippocampus as analyzed by quantitative RT–PCR. GAPDH was used for normalization. (D, E) Protein expressions of NR2B in the hippocampus as analyzed by western blotting. β‐Actin was used as a loading control. (F, G) laparotomy impaired HFS‐elicited LTP in CA1 region in the hippocampus. (H) Verification of virus expression. Bar = 1 mm, left; Bar = 50 μm, right. (I, J) Validation of rLV‐mediated overexpression of NR2B in the hippocampus by western blotting. β‐Tubulin was used as a loading control. (K, L) Average speed (K), and time spent in the central zone (L) of animals in the open field tests. (M) Mean latency of animals in the training phase of the Barnes maze tests (4 consecutive days). (N) Representative tracks (left) and time spent in the target quadrant (right) of animals in the probe phase of the Barnes maze tests. (O) Ratio of freezing time of animals in the contextual fear conditioning tests. The data are presented as the mean ± SD and analyzed by two‐way ANOVA or Student's *t* test. N.S.: no significant difference. *p < 0.05, **p < 0.01, ****p < 0.0001.

To explore the underlying molecular mechanisms, we performed RNA sequencing on the hippocampus from mice that underwent laparotomy and the control mice. Compared to the control mice, we identified 883 differential expressed genes (DEGs) following laparotomy as defined by FDR < 0.05 and fold change > 1.5 with 698 (79.05%) genes were upregulated and 185 (20.95%) genes were downregulated (Figure 2A). Seven neuronal activity‐related genes were included in the downregulated genes (Figure 2B). Among these genes, *Grin2b* encodes the NR2B subunit of NMDA receptors, and NR2B is believed to play a crucial role in synaptic plasticity and memory formation. In line with it, the mRNA level of Grin2b was significant decreased in the hippocampus of the mice that underwent laparotomy (0.54 [0.10] vs. 1.00 [0.30], *p* = 0.0064, Figure 2C).

We then queried whether NR2B is involved in the cognitive decline induced by laparotomy. We first examined the concentration of hippocampal NR2B in mice by western blotting. The results showed that laparotomy significantly decreased the concentration of NR2B (Figure 2D; 0.54 [0.21] vs. 1.00 [0.15], *p* = 0.0405, Figure 2E). Then, we investigated whether laparotomy altered synaptic plasticity with a focus on LTP since previous study reported that NR2B overexpression selectively enhanced LTP rather than long‐term depression (LTD).^27^ As expected, the electrophysiology experiments showed that laparotomy impaired long‐term potential (LTP) in CA1 region in the hippocampus (Figure 2F; 92.13 [13.02] vs. 142.30 [18.25], *p* = 0.0042, Figure 2G). Next, in order to examine the role of NR2B in the pathogenesis of PND, we overexpressed NR2B by bilateral injection of *rLV‐NR2B* into the dorsal hippocampus of aged mice (Figure 2H). Three weeks later, the mice were subjected to laparotomy and then behavioral assessments were performed. To verify the effectiveness of *rLV‐NR2B*, we examined the NR2B concentration by Western blotting. The results showed that the concentration of NR2B in the hippocampus was significantly upregulated with *rLV‐NR2B* injection (Figure 2I; 2.93 [0.61] vs. 1.00 [0.27], *p* = 0.0075, Figure 2J). Additionally, the behavioral results showed that NR2B overexpression had no effect on the locomotor activity of mice that underwent laparotomy but shortened the primary latency in the training phase and prolonged the time spent in the target quadrant in the probe phase of the Barnes maze (Figure 2K–M; 54.23 [4.00] vs. 32.48 [4.33], *p* = 0.0029, Figure 2N). It also increased the ratio of freezing time in the contextual fear conditioning test (44.94 [15.33] vs. 29.28 [10.43], *p* = 0.0357, Figure 2O). These results demonstrate that a decrease in NR2B expression can induce cognitive decline following laparotomy.

### 3.2 Laparotomy increased the methylation levels around the promoter of the *NR2B* gene

**FIGURE 3:**
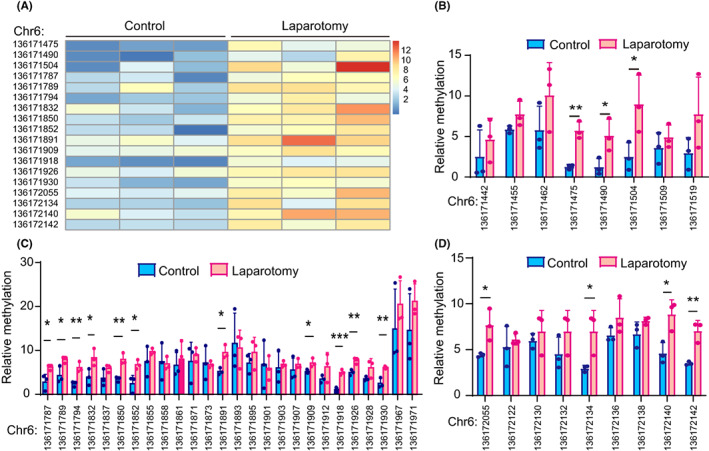
Laparotomy increased the methylation levels around the promoter of the *NR2B* gene in 18‐month‐old mice. (A) Heatmap based on several CpG sites that were significantly hypermethylated in the laparotomy group compared to the Control group. (B‐D) The frequency of methylation at each CpG site in the first (B), second (C), and third (D) predicted CpG islands in the laparotomy group and Control group. The data are presented as the mean ± SD and analyzed by Student's *t* test. *p < 0.05, **p < 0.01, ***p < 0.001.

To determine whether the decreased NR2B expression was attributed to the alteration of *NR2B* gene methylation, we first predicted the CpG islands near the promoter region of the *NR2B* gene using MethPrimer 2.0 (http://www.urogene.org/methprimer2/), and then performed targeted bisulfite sequencing to assess the potential effect of laparotomy on the methylation of the *NR2B* gene promoter in the hippocampus of aged mice. The sequence used for CpG island prediction was shown in Figure S1 (chromosome 6: 136171276–136173094). A total of three potential CpG islands were identified (Chr6: 136171414–136171542, Chr6:136171787–136171978, Chr6:136172055–136172182). The results showed that the methylation level of *NR2B* in mice that underwent laparotomy was higher than that in control mice (Figure 3). Specifically, in the first CpG island, three CpG sites (CpG136171475, CpG136171490 and CpG136171504) showed significant increases in methylation (Figure 3A,B). In the second CpG island, 11 CpG sites (CpG136171787, CpG136171789, CpG136171794, CpG136171832, CpG136171850, CpG136171852, CpG136171891, CpG136171909, CpG136171918, CpG136171926 and CpG136171930) displayed marked increases in methylation (Figure 3A,C). In the third CpG island, four CpG sites (CpG136172055, CpG 136172134, CpG136172140 and CpG136172142) showed obvious increases in methylation (Figure 3A,D). These results indicate that the decreased NR2B expression after laparotomy is likely attributed to the *NR2B* gene hypermethylation.

### 3.3 The increased *NR2B* methylation and decreased NR2B expression were partially attributed to a decrease in SAM concentration

**FIGURE 4:**
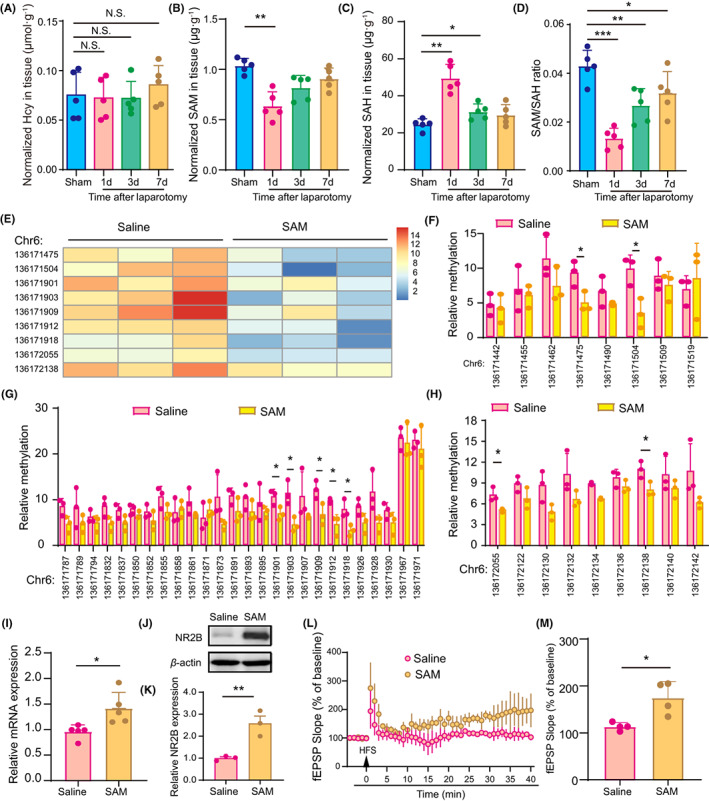
The increased *NR2B* methylation and decreased NR2B expression following laparotomy were partially attributed to a decrease in SAM concentration in 18‐month‐old mice. (A–D) Concentrations of Hcy (A), SAM (B), SAH (C), and SAM/SAH ratio (D) in the hippocampus at the indicated timepoint after the laparotomy. Data in panel (B) are analyzed by Kruskal–Wallis test. (E) Heatmap based on several CpG sites that were significantly hypomethylated in the SAM group compared to the saline group. (F‐H) The frequency of methylation at each CpG site in the first (F), second (G) and third (H) predicted CpG islands in the SAM group and saline group. (I) Levels of NR2B mRNA in the hippocampus as analyzed by quantitative RT–PCR. GAPDH was used for normalization. (J, K) Protein expressions of NR2B in the hippocampus as analyzed by western blotting. β‐Actin was used as a loading control. (L, M) Preoperative SAM treatment improved HFS‐elicited LTP in CA1 region in the hippocampus. The data are presented as the mean ± SD and analyzed by one‐way ANOVA or Student's *t* test unless otherwise specified. N.S.: no significant difference, *p < 0.05, **p < 0.01, ***p < 0.001.

The methylation of DNA can be regulated by one‐carbon (1C) metabolites including Hcy, SAM and SAH.^28^ This prompts us to explore whether these metabolites are affected by laparotomy in the hippocampus of aged mice. Our results showed that the concentration of Hcy was relatively stable (Figure 4A). However, 1 day after laparotomy, the concentration of hippocampal SAM decreased by approximately 36% from 1.02 to 0.65 μg·g^−1^, the concentration of SAH increased by approximately 92% from 24.53 to 47.08 μg·g^−1^, and the SAM/SAH ratio changed from 0.42 to 0.14 (Figure 4B–D). Specifically, the decrease in the SAM/SAH ratio persisted for at least 7 days after laparotomy (Figure 4B–D). These results indicate that a lack of SAM may induce the hypermethylation of the *NR2B* gene.

We next investigated whether preoperative SAM administration could restore the methylation of the *NR2B* promoter. We found that the methylation level of the *NR2B* gene was decreased in the SAM‐treated mice that underwent laparotomy compared to the vehicle‐treated mice that underwent laparotomy (Figure 4E–H). Specifically, in the first CpG island, the CpG sites that showed significant decreases in methylation were CpG136171475 and CpG136171504 (Figure 4E,F). In the second CpG island, the CpG sites that showed marked decreases in methylation were CpG136171901, CpG136171903, CpG136171909, CpG136171912, and CpG136171918 (Figure 4E,G). In the third CpG island, the CpG sites that showed obvious decreases in methylation were CpG 136172055 and CpG136172138 (Figure 4E,H). As predicted, SAM administration increased the expressions of the *NR2B* gene (1.41 [0.32] vs. 0.96 [0.14], *p* = 0.0179) and NR2B protein (2.58 [0.58] vs. 1.00 [0.11], *p* = 0.0097) and improved LTP (175.0 [34.63] vs. 113.3 [8.52], *p* = 0.0135) in the hippocampus of mice that underwent laparotomy (Figure 4I–M). These results indicate that a decrease in SAM concentration is involved in the upregulated *NR2B* methylation and downregulated levels of NR2B mRNA and protein in mice that underwent laparotomy.

### 3.4 Preoperative administration of SAM ameliorated laparotomy‐induced learning and memory decline

**FIGURE 5:**
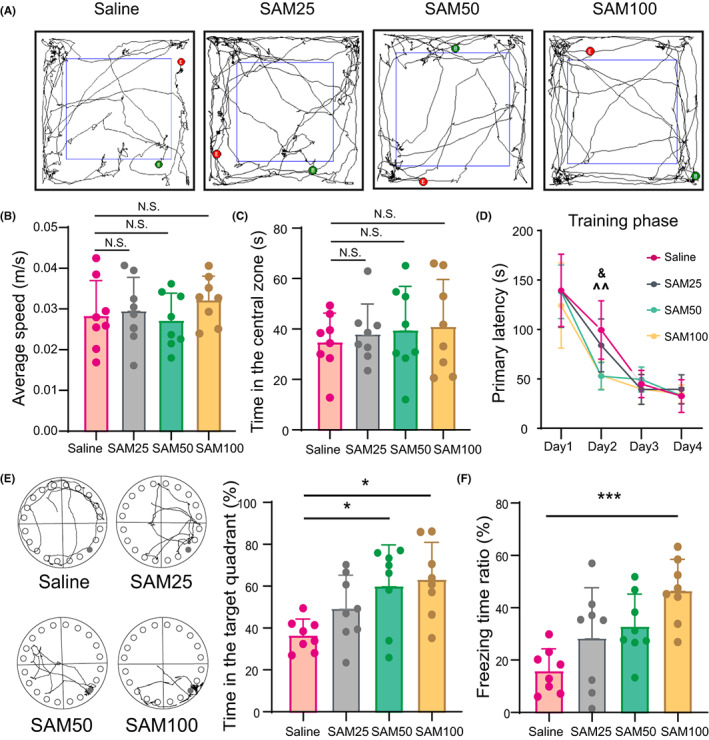
Preoperative administration of SAM ameliorated laparotomy‐induced learning and memory decline in 18‐month‐old mice. (A–C) Representative tracks (A), average speed (B) and time spent in the central zone (C) of animals in the open field tests. (D) Mean latency of animals in the training phase of the Barnes maze tests (4 consecutive days). ^^*p* < 0.01, saline vs. SAM50 group; &*p* < 0.05, saline vs. SAM100 group. (E) Representative tracks (left) and time spent in the target quadrant (right) of animals in the probe phase of the Barnes maze tests. Data are analyzed by Kruskal–Wallis test. (F) Ratio of freezing time of animals in the contextual fear conditioning tests. The data are presented as the mean ± SD and analyzed by two‐way ANOVA or one‐way ANOWA unless otherwise specified. N.S.: no significant difference. *p < 0.05, **p < 0.01, ***p < 0.001.

The results mentioned above prompt us to investigate whether preoperative intraperitoneal injection of SAM is neuroprotective against learning and memory deficits induced by laparotomy. If so, is SAM still protective at lower doses? To answer these questions, aged mice were treated with different doses of SAM (0, 25, 50, 100 mg/kg) 20 min before the laparotomy, and neurocognitive function assessments were performed 3 days later. The results showed that SAM pretreatment did not alter the average speed or time spent in the central zone of mice that underwent laparotomy (Figure 5A–C). However, compared to the control mice that underwent laparotomy, 50 or 100 mg kg^−1^ SAM‐treated mice that underwent laparotomy displayed a shorter primary latency in the training phase (Figure 5D) and spent longer time in the target quadrant in the probe phase in the Barnes maze test (60.07% [19.65] vs. 36.57% [7.687] *p* = 0.01, 63.23% [17.75] vs. 36.57% [7.687] *p* = 0.0032, Figure 5E); however, only mice with 100 mg kg^−1^ SAM exhibited an increased ratio of freezing time in the fear conditioning test (46.48 [12.03] vs. 15.93 [8.351], *p* = 0.0003, Figure 5F). Notably, 25 mg·kg^−1^ SAM‐treated mice that underwent laparotomy did not display any improvement (Figure 5D–F). These results suggest that SAM pretreatment exerts a dose‐dependent therapeutic effect against PND in aged mice.

### 3.5 Inhibiting NR2B abrogated the beneficial effects of SAM against PND

**FIGURE 6:**
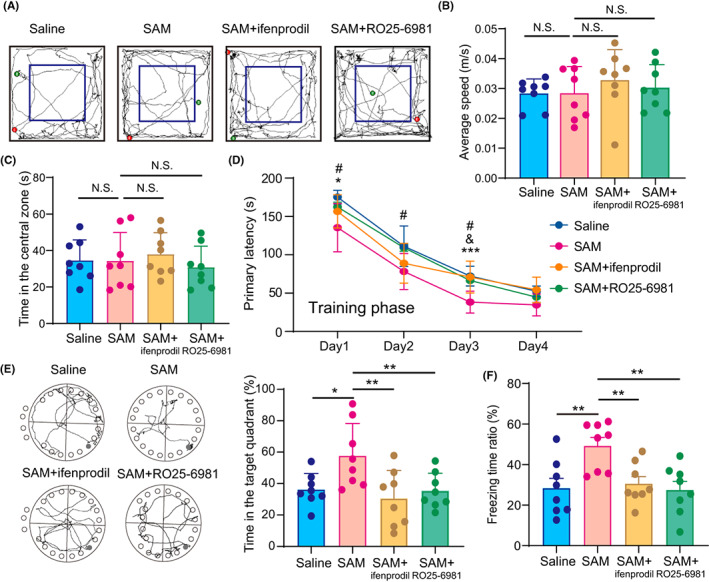
Inhibiting NR2B abrogated the beneficial effects of SAM against PND in 18‐month‐old mice. (A–C) Representative tracks (A), average speed (B) and time spent in the central zone (C) of animals in the open field tests. (D) Mean latency of animals in the training phase of the Barnes maze tests (4 consecutive days). *p < 0.05, ***p < 0.001, SAM versus saline group. &*p* < 0.05, SAM + ifenprodil versus SAM group. #*p* < 0.05, SAM + RO25‐6981 versus SAM group. (E) Representative tracks (left) and time spent in the target quadrant (right) of animals in the probe phase of the Barnes maze tests. (F) Ratio of freezing time of animals in the contextual fear conditioning tests. The data are presented as the mean ± SD and analyzed by two‐way ANOVA or one‐way ANOWA. N.S.: no significant difference. *p < 0.05, **p < 0.01.

To explore the role of NR2B in the therapeutic effect of SAM, the specific NR2B antagonist ifenprodil (10 mg/kg) was intraperitoneally injected into mice treated with SAM (100 mg/kg) three times at 48‐hour intervals starting immediately after the laparotomy, and neurocognitive function assessments were performed. The results demonstrated that ifenprodil treatment did not lead to any locomotor deficit in SAM‐treated mice (Figure 6A–C). However, compared to mice that received only SAM, mice that received both ifenprodil and SAM displayed a longer primary latency in the training phase, spent less time in the target quadrant in the probe phase of the Barnes maze (Figure 6D; 30.53% [17.85] vs. 57.67% [10.21], *p* = 0.0077, Figure 6E), and exhibited a decreased ratio of freezing time in the contextual fear conditioning test (30.56 [9.962] vs. 49.22 [11.82], *p* = 0.0091, Figure 6F). Moreover, mice that received both RO25‐6981 and SAM also displayed a longer primary latency and less time in the Barns maze (Figure 6D; 35.35 [11.25] vs. 57.67% [10.21], *p* = 0.0213 Figure 6E) and exhibited a decreased ratio of freezing time in fear conditioning test (27.43 [12.05] vs. 49.22 [11.82], *p* = 0.0029, Figure 6F). Taken together, these results suggest that inhibiting NR2B abrogates the beneficial effects of SAM against laparotomy‐induced cognitive dysfunction.

## 4 DISCUSSION

In this study, we showed that a decrease in NR2B expression mediated by DNA hypermethylation induced perioperative neurocognitive disorder in aged mice. Preoperative administration of SAM could downregulate the methylation level of the *NR2B* gene promoter, promote the *NR2B* transcription and thereby exhibited a dose‐dependent therapeutic effect against anesthesia and surgery induced cognitive dysfunction.

It has long been recognized that NR2B is involved in the enhancement of learning and memory in mice and that decreased NR2B expression is a main contributor to age‐related cognitive disorders.^29^ In recent years, the importance of NR2B imbalance in PND has been highlighted. However, seemingly discrepant results have been reported across studies. Studies in neonatal and aged rats showed that isoflurane anesthesia alone increased hippocampal and cortical NR2B expression and caused spatial memory deficits.^30^, ^31^, ^32^ Nevertheless, another study showed that liver ischemia–reperfusion under sodium pentobarbital anesthesia decreased hippocampal NR2B expression in adult rats.^33^ We speculated an opposite effect of anesthesia alone and anesthesia‐surgery combination on hippocampal NR2B expression. This hypothesis is partially verified by the current study showing that experimental laparotomy under isoflurane anesthesia decreases hippocampal NR2B expression in aged mice. Although the underlying mechanism is still unknown, these studies demonstrate an important role of NR2B (especially hippocampal NR2B) imbalance in the etiology of PND. Surprisingly, another study in 9‐month‐old mice reported that synaptic NR2B in the hippocampus contributed little to cognitive decline induced by a surgical incision under isoflurane anesthesia.^34^ These discrepancies might be explained by differences in the age of the mice used, the anesthesia and/or surgery and the region in which the NR2B expression was examined. Further studies are necessary to elucidate the complicated role of hippocampal NR2B in the pathogenesis of PND.

The expression of NR2B has been reported to be negatively regulated by DNA methylation. For example, a decreased methylation level of the *NR2B* gene corresponds to increased NR2B mRNA and protein expressions in mice following chronic ethanol treatment,^12^ and an increase in the methylation level of the *NR2B* gene corresponds to decreased NR2B mRNA and protein expressions in mice with kainic acid‐induced epilepsy or benzo(a)pyrene (BaP)‐induced cognitive impairment.^13^, ^35^ Consistent with these observations, we showed that upregulated methylation of the *NR2B* gene corresponds to decreased *NR2B* transcription in mice subjected to laparotomy, adding evidence that supports the regulatory role of DNA methylation in *NR2B* transcription. Additionally, it is worth noting that the methylation level of several CpG sites in the *NR2B* gene, including CpG136171475, CpG136171504, CpG136171909, CpG136171918, and CpG136172055 on Chromosome 6, is highly correlated with cognitive performance, implicating that the methylation levels at these sites may be utilized as biomarkers for PND diagnosis. Though, NR2B promoter hypermethylation as a cause or an effect is not conclusive, expressing *NR2B* gene that harbors mutations in these CpG sites in 18‐month‐old NR2B deficient (KO) mice would help solve this problem. We speculated that these sites are essential for the binding of proteins that regulate the transcription of *NR2B*. Previous studies have reported that the mouse *NR2B* gene harbors three non‐coding exons in the 5′UTR and that the exon 2 of the *NR2B* gene contains many regulatory sequences, such as a cyclic AMP‐responsive element (CRE)‐binding site and Sp1 (a transcription factor)‐binding site.^36^, ^37^ Further chromatin immunoprecipitation (ChIP) assays are needed to clarify the relationships between *NR2B* gene methylation and the accessibility of these two transcription factors and their roles in the pathogenesis of PND.

To examine the potential mechanism by which laparotomy upregulates the methylation of the *NR2B* gene, we investigated the concentrations of one‐carbon metabolites since they were crucial in regulating global DNA methylation by determining the flux of methyl groups. We found that the concentration of Hcy was relatively stable, which was consistent with our previous study.^15^ Additionally, we found a significant decrease in the SAM/SAH ratio, an indicator of global DNA methylation, which is likely to contradict the increased *NR2B* methylation observed after laparotomy. However, similar results were observed in the processes of aging and carcinogenesis.^9^, ^38^ Indeed, studies on PND have also observed global DNA hypomethylation,^11^, ^39^ and DNA hypermethylation of several genes (e.g. *glucocorticoid receptor, Arc, Bdnf, Reln, PSD‐95*),^10^, ^40^, ^41^ indicating that the global DNA methylation status did not necessarily reflect the methylation of specific genes. More importantly, we found that SAM could function as a “hypomethylating” agent to hypomethylate the *NR2B* gene, supporting that SAM is a methylome modulator rather than a pure “hypermethylating” agent as it used to be predicted.^42^ We speculate that SAM might facilitate the dissociation of DNA methyltransferase (e.g. Dnmt1, Dnmt3a/b) from the *NR2B* gene or the binding of DNA demethylase (e.g. 10–11 translocation, TET1, TET2) to the *NR2B* gene.

As a complementary and integrative medicine, SAM is widely available and has been approved for clinical use for the treatment of several neuropsychiatric disorders for several decades. Particularly, SAM deficiency in the cerebrospinal fluid has been reported in patients with Alzheimer's disease and Parkinson's disease.^43^, ^44^ Importantly, previous studies demonstrated that SAM had little effect on the healthy/control animals,^45^, ^46^, ^47^ although we did not verify this in this study, our results clearly showed that SAM monotherapy at 100 mg/kg was sufficient to improve cognitive outcomes after anesthesia and surgery. Based on a simple practice guide for dose conversion, using 100 mg/kg SAM in mice approximates using 500 mg in adults weighed 60 kg,^48^ which is much less than 1600 mg/day used in clinic,^49^ indicating that our research has potential translational value for PND prevention in elderly patients who are susceptible to PND. Regrettably, only male mice were used in this study. Previous studies demonstrate that sexual dimorphism occurs in brain structure and gene expression, cerebral blood flow and metabolism, and cognitive and psychiatric disorders in rodent animals,^50^, ^51^, ^52^ the potential role of SAM in female mice is worth being investigated in future.

## 5 CONCLUSION

In conclusion, our study suggests that the hypermethylation of *NR2B* gene, which partially results from SAM deficiency, can induce decreased hippocampal NR2B expression and hippocampus‐dependent cognitive deficits following laparotomy. These findings may provide new preventive and therapeutic approaches for cognitive disorders induced by laparotomy.

## AUTHOR CONTRIBUTIONS

Lize Xiong and Feifei Xu designed and supervised the research. Feifei Xu and Peilin Cong conducted the experiments and wrote the manuscript. Bingqian Zhang performed the electrophysiological recording. Hailong Dong, Wenqiang Zuo, Tingmei Wu and Li Tian helped in revising the manuscript. All authors reviewed the results and approved the final manuscript.

## FUNDING INFORMATION

This study was supported by grants from the National Natural Science Foundation of China (81901078, 82271212 to F.X.), the Key Project of National Natural Science Foundation of China (81730032 to L.X.), the Science and Technology Commission of Shanghai Municipality (201,409,003,500 to L.X.), and the National Natural Science Foundation of China (81870824 to L.T.).

## CONFLICT OF INTEREST

The authors have declared that no conflict of interest exists.

## Supporting information


Table S1

Figure S1

## Data Availability

The data that support the findings of this study are available from the corresponding author upon reasonable request.
