# Supplementary material for: A decrease in NR2B expression mediated by DNA hypermethylation induces perioperative neurocognitive disorder in aged mice
Source: CNS Neurosci Ther. 2023 Jan 24;29(5):1229–42. doi: 10.1111/cns.14097 (PMC10068472; doi:10.1111/cns.14097)
Supplement: Supplementary file 1 — Table S1 [file CNS-29-1229-s001.docx]

**Table 1. Primers used for targeted bisulfite sequencing.**

| **Primers used for PCR #1** | **Sequence** |
| --- | --- |
| PCR_F1 | TCCCTACACGACGCTCTTCCGATCTGGTTTGGTTTTATGGAGAAG |
| PCR_R1 | AGTTCAGACGTGTGCTCTTCCGATCTAAACTTCRAACCTTTATCTACCT |
| PCR_F2 | TCCCTACACGACGCTCTTCCGATCTGGATAGGATTGTTTTTGGTTT |
| PCR_R2 | AGTTCAGACGTGTGCTCTTCCGATCTATACACCRACCRCACACTAAA |
| PCR_F3 | TCCCTACACGACGCTCTTCCGATCTTGTTAGTGTGTTTTATTTAAGAAATG |
| PCR_R3 | AGTTCAGACGTGTGCTCTTCCGATCTAAACTCCACACAACAATTCAC |
| PCR_F4 | TCCCTACACGACGCTCTTCCGATCTTTTAGGAAYGGTATAGGTAGATTT |
| PCR_R4 | AGTTCAGACGTGTGCTCTTCCGATCTATCAAAATTTAATTCRAATATCTCC |
| PCR_F5 | TCCCTACACGACGCTCTTCCGATCTGGAGATATTYGAATTAAATTTTGAT |
| PCR_R5 | AGTTCAGACGTGTGCTCTTCCGATCTAAACCATTATTCCATATACATATAAA |
| PCR_F6 | TCCCTACACGACGCTCTTCCGATCTTTTGGATTTTGTATTGTGAGT |
| PCR_R6 | AGTTCAGACGTGTGCTCTTCCGATCTCCTACCTTAATTTATAAAAACCAA |
| **Primers used for PCR #2** | **Sequence** |
| i5 Index Primers | AATGATACGGCGACCACCGAGATCTACAC[i5]ACACTCTTTCCCTACACGACGCTCTTCCGATCT |
| i7 Index Primers | CAAGCAGAAGACGGCATACGAGAT[i7]GTGACTGGAGTTCAGACGTGTGCTCTTCCGATCT |
| **I5 index** | **I7 index** |
| ATCGGCTT | CTGGACTT |
| TGCACTCA | AGCAGACA |
| CCTTAGAC | CGGTACTA |
| GAAATCGG | TGCAATGC |
| CTGCATGA | AATTGCAT |
| TTGCATCT | TTTACCGT |
| ACGTGGAC | CTGGAACC |
| GACTTGAG | TGGGACTC |
