# Supplementary material for: A decrease in NR2B expression mediated by DNA hypermethylation induces perioperative neurocognitive disorder in aged mice
Source: CNS Neurosci Ther. 2023 Jan 24;29(5):1229–42. doi: 10.1111/cns.14097 (PMC10068472; doi:10.1111/cns.14097)
Supplement: Supplementary file 2 — Figure S1 [file CNS-29-1229-s002.docx]

**Supplementary Material for Review**


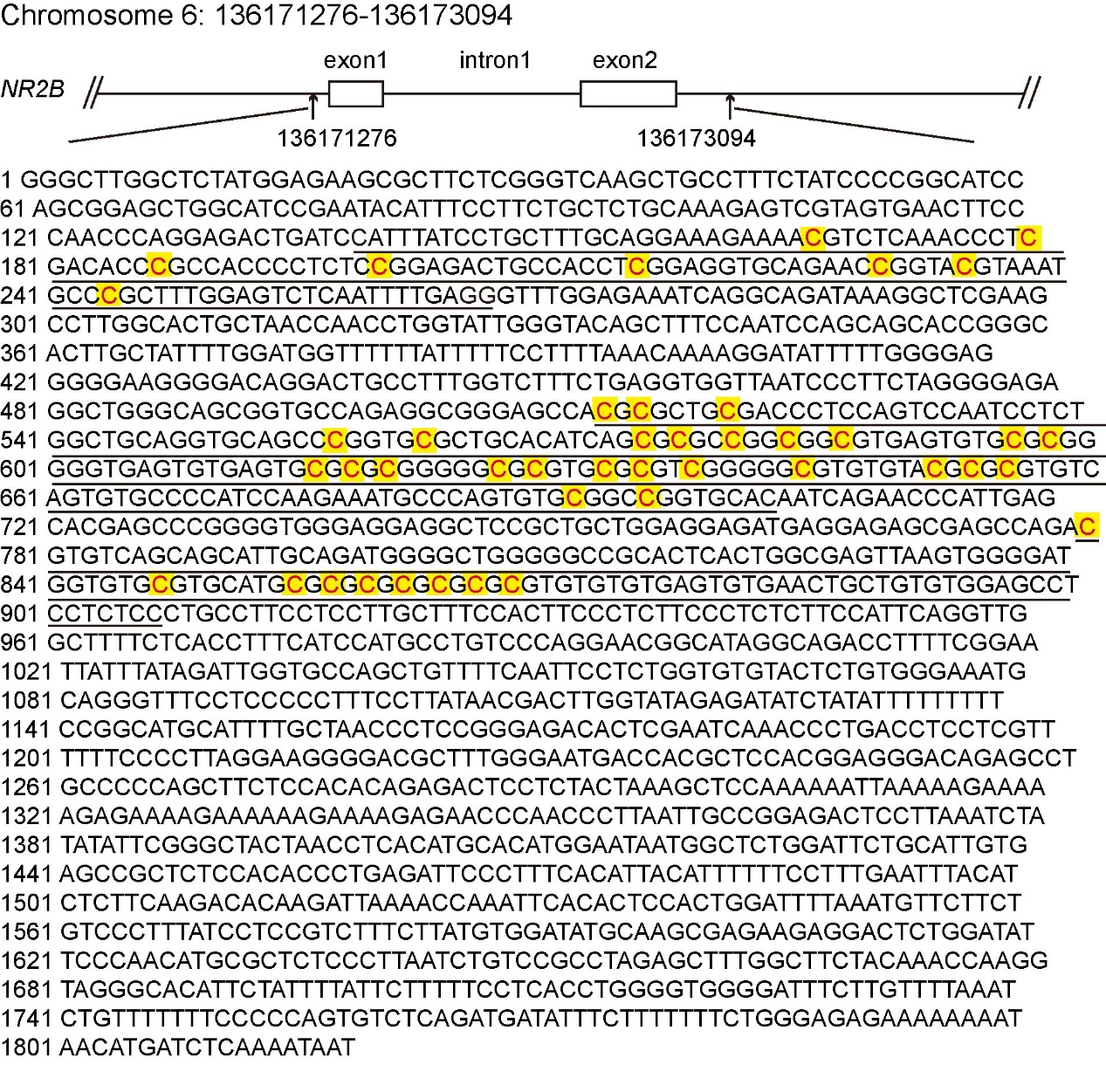


**sFig. 1 Prediction of CpG islands in the promoter of the *NR2B* gene. The three predicted CpG islands are underlined and the CpG sites are highlighted in yellow.**


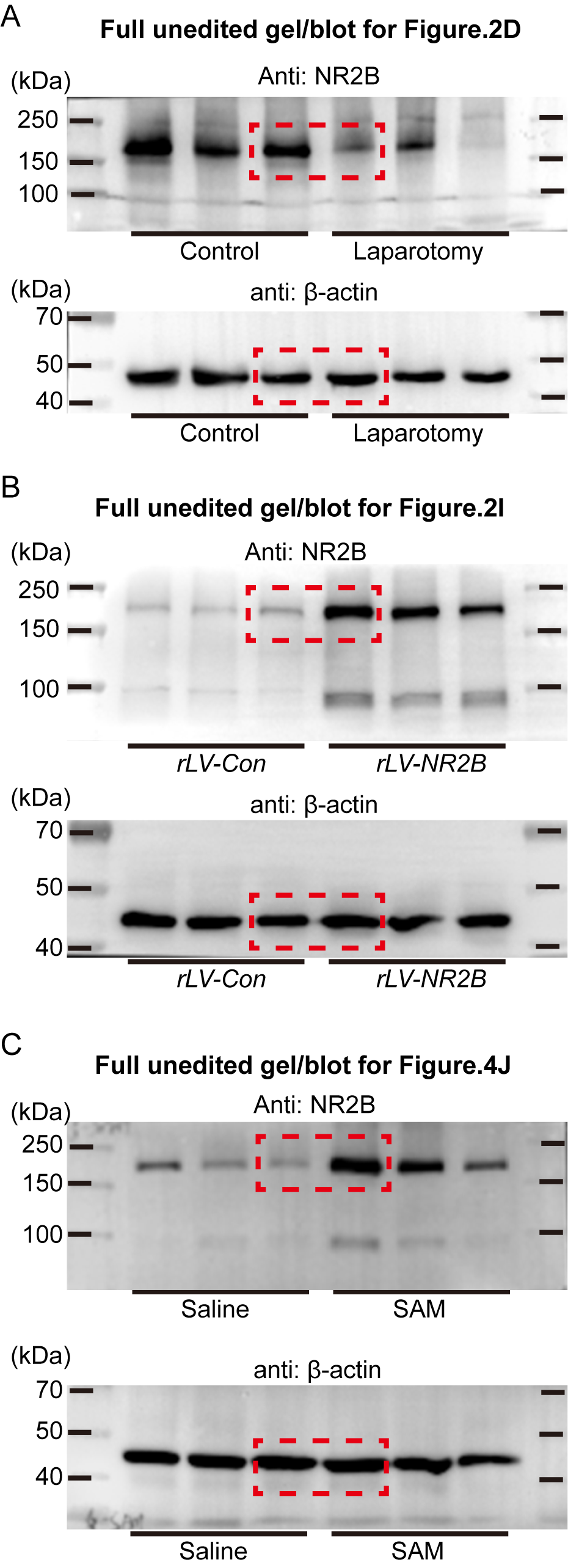


**sFig. 2 Full unedited gel/blot in the article.**
